# Impact of communities, health, and emotional-related factors on smoking use: comparison of joint modeling of mean and dispersion and Bayes’ hierarchical models on add health survey

**DOI:** 10.1186/s12874-017-0303-y

**Published:** 2017-02-03

**Authors:** Jie Pu, Di Fang, Jeffrey R. Wilson

**Affiliations:** 1 School of Mathematical and Statistical Science, Arizona State University, Tempe, USA; 2 Department of Agricultural Economics and Agribusiness, University of Arkansas, Fayetteville, USA; 3 Department of Economics, Arizona State University, Tempe, USA

**Keywords:** Overdispersion, Generalized additive models, Generalized linear models, Bayes’ hierarchical model, Logistic regression

## Abstract

**Background:**

The analysis of correlated binary data is commonly addressed through the use of conditional models with random effects included in the systematic component as opposed to generalized estimating equations (GEE) models that addressed the random component. Since the joint distribution of the observations is usually unknown, the conditional distribution is a natural approach. Our objective was to compare the fit of different binary models for correlated data in Tabaco use. We advocate that the joint modeling of the mean and dispersion may be at times just as adequate. We assessed the ability of these models to account for the intraclass correlation. In so doing, we concentrated on fitting logistic regression models to address smoking behaviors.

**Methods:**

Frequentist and Bayes’ hierarchical models were used to predict conditional probabilities, and the joint modeling (GLM and GAM) models were used to predict marginal probabilities. These models were fitted to National Longitudinal Study of Adolescent to Adult Health (Add Health) data for Tabaco use.

**Results:**

We found that people were less likely to smoke if they had higher income, high school or higher education and religious. Individuals were more likely to smoke if they had abused drug or alcohol, spent more time on TV and video games, and been arrested. Moreover, individuals who drank alcohol early in life were more likely to be a regular smoker. Children who experienced mistreatment from their parents were more likely to use Tabaco regularly.

**Conclusions:**

The joint modeling of the mean and dispersion models offered a flexible and meaningful method of addressing the intraclass correlation. They do not require one to identify random effects nor distinguish from one level of the hierarchy to the other. Moreover, once one can identify the significant random effects, one can obtain similar results to the random coefficient models. We found that the set of marginal models accounting for extravariation through the additional dispersion submodel produced similar results with regards to inferences and predictions. Moreover, both marginal and conditional models demonstrated similar predictive power.

**Electronic supplementary material:**

The online version of this article (doi:10.1186/s12874-017-0303-y) contains supplementary material, which is available to authorized users.

## Background

The standard logistic regression model is commonly used in the analysis of uncorrelated binary response observations with several covariates [1]. It is a member of the generalized linear models (GLM) where the canonical link is the logit, and the random component is binomial. Its ability to appeal to the odds of occurrence of an event has gained great interest and popularity in fitting binary data. However, when correlated observations are modeled with a standard logistic regression, the results are not necessarily efficient or reliable. In such cases, we can use the generalized estimating equation (GEE) model or a generalized linear mixed model (GLMM), or as emphasized in this paper, resort to the joint modeling of the mean and the dispersion. This additional modeling of the dispersion (beyond the generalized linear model for the mean) allows us to correct for the inflated standard errors due to the intraclass correlation and thereby provides more reliable and efficient estimates. The additional submodel has other benefits, as there are cases when one may be only interested in modeling the dispersion. Smyth and Verbyla [2] among others proposed joint modeling of the mean and dispersion parameters for certain distributions. Taylor and Verbyla [3], Wu, Zhang and Xu [4] proposed a unified procedure for selecting significant covariates in the mean and the dispersion sub models.

In survey research, one often encounters correlated data due to the designed nature of the study, longitudinal data or clustered data. Such data usually referred to collectively as repeated measures, often emanates in healthcare, education, psychology, sociology, and other related areas. As an example, in an obesity study among adolescents, students were sampled from within schools, and schools were sampled from within school districts. In such a situation, the observations were correlated due to the hierarchical structure of the design. Thus, to fit standard logistic regression models to such data will require one to violate of the usual assumption of independence. As such one may declare covariates as significant when in fact, they are not. In fact, it is not appropriate to model correlated data with any generalized linear model, as such models assume that the dispersion parameter is fixed [5]. In other words, the generalized linear model addresses location as a centering of the data, but the spread is not assumed to vary. However, in most of these cases when the distribution of response distribution belongs to the exponential family the spread is often related to the mean. On the other hand, it is well known that in most cases in the analysis of correlated data, that fixed dispersion or dispersion tied directly to the mean is less common than one assumes when confronted with survey data. However, the joint modeling of the mean and the dispersion is an approach where covariates are used to model both the mean and the dispersion simultaneously. In fact, the estimators of the regression parameters in the mean submodel is more efficient when the dispersion is correctly addressed [6].

In this paper, we modeled smoking based on a set of covariates. We compared different approaches to modeling these correlated data. These methods included marginal models based on the joint modeling of the mean and the dispersion and conditional models based on the hierarchical structure of the data with the use of random effects. The basis for the joint modeling is to directly address the correlation in the data [6]. In this paper, the joint modeling of the mean and the dispersion includes the generalized linear model (GLM) and generalized additive model (GAM). These are marginal models.

We fitted, compared, and validated several logistic regression models as we modeled smoking, as defined by having at least one cigarette every day for 30 days. Smoking is detrimental as it can cause lung cancer, COPD (chronic obstructive pulmonary disease), heart disease, stomach cancer, as well as sudden infant death syndrome for women. Studies related to smoking have found that race and gender accounted for a differential role in the regular smoking habit of adults, while other studies have found an interactional effect between smoking, drinking, and income [7]. One study found that advertisements through social media such as movies and television have been linked with adolescent smoking initiation [8]. Yet another study found that patients with mental illness have a higher incidence of smoking than the general population [9].

### Data

We obtained 4,484 observations from the fourth wave of Add Health survey [10], which was initially designed based on a two-level clustered design with an in-home questionnaire survey. Respondents were selected randomly from 132 different communities and were followed from 1995 to 2009. We selected variables based on social demographics, health status, and psychological status. A description of chosen predictors is presented in Table 1.

**Table 1:** Description of variables in our study

	Explanation
Demographic	Do you have degree above high school?
	What was the total household income before taxes and deductions?
	How many of these children are still living?
	Have you ever been Arrested?
	Gender
	Are you white?
Health	Do you have health insurance?
	How long ago did you last have a routine check-up?
	Do you have more than 5 drinks or drink alcohol more than 3 days a week during the past 12 months?
	Do you have prescription drug misuse?
	On the average, do you use a physical fitness or recreation center in your neighborhood per week?
	In the past seven days, how many hours did you watch television or videos, including VHS, DVDs or music videos?
	How old were you when you first had an Alcoholic drink?
Emotional	How important (if at all) is your religious faith to you?
	Before your 18th birthday, did a parent or other adult caregiver say things that really hurt your feelings or made you feel like you were not wanted or loved or hit you with a fist, kick you, or throw you down on the floor, into a wall, or down stairs more than 10 times?
	In the past 12 months, did you spend on volunteer or community service work?
	Are you optimistic about your future?
	Do you feel isolated from others?
	Do you satisfy with your current job?

This sample consisted of respondents aged between 25 and 34 years, with 46.01% being male, and 32.4% being non-Caucasians. Approximately 45.2% of the respondents smoked cigarettes regularly (that is, at least one cigarette every day for 30 days), whereas 75% drank socially. 81% had both smoked and drunk at least once. Among those individuals who had both used alcohol or smoked, 20.9% had experienced smoking first, 23.1% had experienced alcohol first and 12.5% first experienced smoking and drink alcohol at the same time. On the health aspect, 37.5% of the sample had their last routine checks more than one year ago, 21.4% had no health insurance, and 30.6% had routine sports time. Regarding emotional satatus, 10.3% had psychological consulting experience, 13.5% had childhood mistreatment experience from their parents, 29.1% had negative or neutral attitude towards their future, and 28.8% felt isolated from the social life. Job satistfaction was average d at 27.7%.

## Methods

We started with an unconditional hierarchical logistic regression model to obtain the intraclass correlation coefficient (ICC)^1^. The ICC measures the closeness of the individuals within a cluster as it pertains to smoking [11]. We found that ICC to be sufficiently large to warrant a correlated model [12]. We then conducted an analysis of smoking based on four (two marginal and two conditional) models appropriate for correlated binary data. We identified and addressed similarities and differences with these models as they addressed and accounted for the intraclass correlation. The two hierarchical models, one frequentist and one Bayes, and the two joint mean and dispersion models, one generalized linear model (GLM) and the other generalized additive model (GAM), were utilized. We compared the fit of these models as they pertained to their predictive ability on smoking. We stayed clear of addressing their mathematical or computational differences [13]. Our comparisons were made based on withholding a portion of the data for validation. We randomly selected 75% for the training dataset and 25% for validation dataset. We did this four different times, choosing a different 25% each time.

### Hierarchical logistic regression models

We presented two conditional logistic regression or hierarchical logistic regression models for analysis. The dependency among observations was accounted for through adjustment to the systematic component with the addition of random intercepts and random slopes. This method of adjustment to the systematic component requires additional distributional assumptions as it now consists of random effects in addition to the fixed effects. This approach results in modeling the conditional mean rather than the marginal mean, thus, are referred to as subject-specific models [14]. Essentially, the subject-specific model consists of a product of a set of distributions, one set based on the conditional mean given the random effects and the other for the assumed distribution of the random effects. In our example, a subject -specific model tells us about the probability that the individual smoked given the community (random effect) [13].

Consider modeling smoking as the outcome as a Bernoulli random variable Y_ij_, which takes on the value of one (if smoking) with probability P_ij_ and covariates X_ijk_ for the i^th^ individual in j^th^ community for the k^th^ covariate, i = 1,2,....I; j = 1,2,....J; k = 1,2,.....K. Let the overall differential cluster random effects be denoted as γ_0j_(random intercept in the model) and assumed to be distributed as a normal random variable with mean zero and variance δ_0_^2^. This addresses the unmeasured impact of the communities on the individual. The random intercept γ_0j_ represents the combined effect of all omitted covariates that causes individuals to be more prone to smoking. Our initial exploration of the data and some complimentary research suggested a differential effect across communities with regard to *arrest*. Thus, let the coefficient for *arrest* show a differential rate from community to community (random slope in the model). Random slope is denoted by γ_1j_ and assumed to be distributed normally with mean zero and variance δ_1_^2^. Finally, the hierarchical logistic regression model with $19$ covariates and random intercept and random slope is:

1 $$ \mathrm{logit}\ \left({\mathrm{p}}_{\mathrm{ij}}\ \Big|\ {\mathrm{X}}_{\mathrm{ij}\mathrm{k}}\ {\upgamma}_{0\mathrm{j}},\ {\upgamma}_{1\mathrm{j}}\right) = {\upbeta}_0+{\upbeta}_{1\ }{\mathrm{X}}_{\mathrm{ij}1} + {\upbeta}_{2\ }{\mathrm{X}}_{\mathrm{ij}2}+\cdots + {\upbeta}_{\mathrm{K}\ }{\mathrm{X}}_{\mathrm{ij}\mathrm{K}}+\kern0.5em {\upgamma}_{0\mathrm{j}} + {\upgamma}_{1\mathrm{j}}\ {\mathrm{Z}}_{\mathrm{ij}1} $$

where X_ijk_ represents the k^th^ covariate measure for the i^th^ person in the j^th^ cluster (community) β_i_ are the regression coefficients associated with covariate X_ijk_ β_0_ is the overall fixed effects and Z_ij1_ is the covariate *arrest* in this case associated with the random slope coefficient of arrest, with conditional distribution Y_ij_| X_ijk_ γ_0j_, γ_1j_ ~ *Bin* (n_i_, p_ij_) where *Bin* denotes the binomial distribution, the random intercept for communities γ_0j_ ~ *N*(0, δ_0_^2^) and random slope γ_1j_ ~ *N*(0, δ_1_^2^) where *N* denotes the normal distribution and the correlation of the random effects intercept and slope parameters is σ_γ0j,γ1j_. The correlation is due to the fact that observations in the same cluster share similar effects. The random slope γ_1j_ represents that there are differential rates of change with subjects in each cluster (community) as it relates to the covariate *arrest* Z_ijk_. We referred to this as frequentist hierarchical logistic regression model as opposed to the Bayes’ hierarchical logistic regression model with its additional set of prior distributions on the parameters. We fitted the frequentist hierarchical model with PROC GLIMMIX in SAS, which is presented in Additional file 1: Appendix.

### Bayes’ hierarchical logistic regression model

Bayesian hierarchical logistic regression model is presented as:

2 $$ \mathrm{logit}\ \left({\mathrm{p}}_{\mathrm{i}\mathrm{j}}\ \Big|\ {\mathrm{X}}_{\mathrm{i}\mathrm{j}\mathrm{k}}\ {\upgamma}_{0\mathrm{j}},\ {\upgamma}_{1\mathrm{j}}\right) = {\upbeta}_0+{\upbeta}_{1\ }{\mathrm{X}}_{\mathrm{i}1} + {\upbeta}_{2\ }{\mathrm{X}}_{\mathrm{i}2}+\cdots + {\upbeta}_{\mathrm{K}\ }{\mathrm{X}}_{\mathrm{i}\mathrm{K}}+\kern0.5em {\upgamma}_{0\mathrm{j}} + {\upgamma}_{1\mathrm{j}}\ {\mathrm{Z}}_{\mathrm{i}\mathrm{j}1} $$

differs from the frequentist hierarchical model [1] merely in the set of prior distributional assumptions attached to the parameters in the Bayes’ logistic regression model. The Bayes’ logistic regression model requires that there are distributional assumptions specified for the unknown β_i_ parameters, as well as the covariance parameters δ_0_^2^ δ_1_^2^ and σ_γ0j,γ1j_ associated with the distribution for the random effects γ_0j_, γ_0j_ ~ *N*(0, δ_0_^2^) and γ_1j_ the random slope γ_1j_ ~ *N*(0, δ_1_^2^) respectfully. The β’s are assumed to be normally distributed. The Bayes’ model requires that we incorporate any prior information on the unknown parameters, **β** = β_0_, β_1_, …, β_K_; δ_0_^2^ , δ_1_^2^ and σ_γ0j,γ1j_ together with the information we obtained from the observed data. Thus we concentrate on the resulting posterior distribution from which we seek posterior modes. In fact, the prior knowledge is represented through the distributional assumptions. It allows updating the knowledge regarding the unknown parameter distribution as if its prior information is known. We used the prior information through the distribution Pr(**β**) which is based on initial beliefs to estimate and make inferences. It is customary to assume that the normal distribution is the most appropriate prior. Thus, we obtained the posterior distribution, as proportional (∝) to the product of the likelihood function and the prior distribution on, which we concentrate. So that the probability is followed through the posterior,

3 $$ \Pr\ \left[\boldsymbol{\upbeta} \kern0.75em {\upgamma}_{0\mathrm{j}},\ {\upgamma}_{1\mathrm{j}},\ {\updelta}_0^2,{\updelta}_1^2\Big|\mathrm{Y}\right] \propto \left[ \Pr \left[\mathrm{Y}\Big|\boldsymbol{\upbeta},\ {\upgamma}_{0\mathrm{j}},\ {\upgamma}_{1\mathrm{j}},\ {\updelta}_0^2,{\updelta}_1^2\right]\ast \Pr \left(\boldsymbol{\upbeta},\ {\upgamma}_{0\mathrm{j}},\ {\upgamma}_{1\mathrm{j}},\ {\updelta}_0^2,{\updelta}_1^2\right)\right] $$

Since the data Y have distribution, Pr(Y) as constant relative to **β**. If the prior distributions were chosen to be uniform instead of normal, then the estimates for equations [1] and [2] are equivalently both maximum likelihood estimates. We obtained estimates from the posterior distribution, [2.3]. Such posterior inference typically requires simulation techniques such as Gibbs sampling and the Metropolis Hasting sampling as a MCMC method to obtain estimates. In fact, it generates new values from a proposed distribution that determines how to select new parameter values based on the current values. The Bayesian procedure produces consistent and efficient estimates. It has also been shown that if we were to choose conjugate priors, it guarantees that a posterior distribution possesses the same property as the prior distribution [15]. We used MLWin to fit these Add Health data with Bayes’ hierarchical logistic regression model. MLwiN is a powerful program designed for fitting multilevel models. It provides features such as *variance function window* to calculate the residual variance at any level.

### Joint modeling of the mean and dispersion

The subject-specific models concentrated modeling the conditional mean through the use of random effects to address the correlation. However, there is added value to address the correlation through jointly modeling of the mean and the dispersion [7]. This joint modeling consists of two interlinked models, one for the mean and one for the dispersion [16]. This differs from the random coefficient models and its approach to addressing correlation. It is a reflection of the double exponential family that allows us to model the mean parameter while making use of a second parameter that addresses the variance but independently of the mean [17]. Each submodel is considered to be a generalized linear model with three components: random component, systematic component, and link component [18]. The joint modeling of the mean and dispersion consists of starting initially with the mean submodel. Then, the residuals from the mean submodel or a function of them are used as the response (random component) in the dispersion submodel. The systematic component in the dispersion submodel is allowed if needed, to have the same or a subset of covariates as in the mean submodel or a totally different set of covariates not yet used. The link function in the dispersion submodel follows similar procedure though not necessarily the same function as the mean submodel. We used the same model checking techniques for both the mean and the dispersion submodel [19]. We considered two different joint modeling of both the mean and the dispersion submodel (Double Joint Modeling Mean and Dispersion), one based on GLM and the other based on GAM.

### Double GLM Joint Modeling mean and dispersion

The joint modeling of the mean and the dispersion, also referred to as double generalized linear models (DGLM) consists of three parts: a function for the variance, a GLM submodel for the mean, and a GLM submodel for the dispersion [2]. Thus, we need to obtain an estimation of the mean function and estimation of the variance function [20].

In particular, let us assume that the binary random variable Y_i_ is Bernoulli with probability ${\mathrm{p}}_{\mathrm{i}}$, which depends on a set of covariates **X** = (X_1_,X_2_, … X_K_). The mean submodel is first fitted with logit link. The deviances from the mean submodel ${\mathrm{d}}_{\mathrm{i}}$ have mean ϕ_i_ with variance V_di_ (ϕ_i_) representing the random component. The systematic component of the dispersion submodel consists of the vector of covariates **Z** = (Z_1_, Z_2_, … Z_t_) with link component as log. In our model we have one covariate in the dispersion submodel as *arrest*. In summary, we have two interlinked generalized linear models consisting of the mean submodel, measuring smoking use *Yi ~ Ber*(*p*_*i*_), where *p*_*i*_ represents probability of smoking with logit link,

4 $$ {\upeta}_{\mathrm{i}} = \mathrm{logit}\ \left({\mathrm{p}}_{\mathrm{i}}\right) = \log \left(\frac{{\mathrm{p}}_{\mathrm{i}}}{{1\hbox{-} \mathrm{p}}_{\mathrm{i}}}\right) = {\upbeta}_0 + {\upbeta}_1{\mathrm{X}}_1+\cdots +{\upbeta}_{\mathrm{K}}{\mathrm{X}}_{\mathrm{K}} $$

and the dispersion submodel based on ${\mathrm{d}}_{\mathrm{i}}$ such that d_i_ ~ D_d_ (ϕ_i_,V_di_(ϕ_i_)) and log link

$$ {\mathrm{n}}_{\mathrm{di}} = \log \left({\upphi}_{\mathrm{i}}\right) = {\upgamma}_0 + {\upgamma}_1{\mathrm{Z}}_1. $$

We used PROC QLIM and Macro HPGLIM in SAS to fit these models, as presented in Additional file 1: Appendix.

### Double GAM Joint Modeling mean and dispersion

In the joint modeling of the mean and dispersion one can replace those GLM with GAM. As such we still present two submodels, one for mean and one for the dispersion, but in each we have a generalized additive model instead of GLM. A general additive model is similar to generalized linear model as it relates the mean of the random response variable Y and a set of covariates X_i_, …, X_p_, [21]. The generalized additive model (GAM) distinguishes itself from the generalized linear model in that

5 $$ \mathrm{E}\left[\mathrm{Y}\right] = \kern0.5em {\upgamma}_0+\kern0.5em {\upgamma}_1\left({\ \mathrm{X}}_1\right)+\dots + {\upgamma}_K\left({\ \mathrm{X}}_K\right) $$

where γ_i_ (X_i_), i = 1, …,K; are smooth functions. These smooth functions γ_i_, are not given a parametric form but instead are estimated in a nonparametric or semi-parametric form. The GAM consists of a random component with response Y_i_, a systematic component that is additive in the function of covariates, and a link function relating the response in terms of the mean with a combination of the covariates. Whereas GLM has as its systematic component the linear predictor of the form Σβ_i_X_i_ the GAM instead, uses the additive component, as a sum of smooth functions Σγ_i_(X_i_). The function γ_i_ is determined or estimated based on a nonparametric technique usually influenced by the examination of actual plots. The GAM requires one to determine the smoothing parameter through the generalized cross validation (GCV) function in nonparametric regression methods [22]. The functions γ (⋅) and f (⋅) are additive smooth functions, unspecified linear functions or non-parametric methods, for both mean and dispersion submodels.

The GAM is known to be more flexible than the parametric methods as an exploratory analysis in identifying the relationship between the response and the covariates. Also it is more flexible as opposed to other models when detecting quadratic or higher order power in a piecemeal fashion [21]. In addition, the GAM can also be used more efficiently to uncover nonlinear effects among the covariates. While the generalized linear model is used for estimation and inferences for the regression parameters, the GAM is seen as an exploratory method based on a nonparametric approach while making use of an apparent response-covariate relationship. In summary, we have the systematic component for the mean additive submodel logit (p_i_) = ∑_i = 1_^p^γ_i_(X_i_) and the systematic component for dispersion additive submodel log(deviance_i_) = ∑_i = 1_^k^f_i_(X_i_). The log (deviance) is assumed to be distributed as a normal distribution in the dispersion with identity link function [23]. We used PROC GAM in SAS to fit these models, as presented in Additional file 1: Appendix.

## Results

We fitted two logistic regression models with random effects, frequentist hierarchical logistic regression model and Bayes’ hierarchical logistic regression model. We also fitted two joint modeling of the mean and dispersion models, one based on the generalized linear model, and the other on generalized additive modeling. Results of all models are presented in Table 2.

**Table 2:** Parameter estimates and standard errors in hierarchical logistic regression

Estimates	Hierarchical logistic		Bayes’ Hierarchical logistic		Joint GLM		Joint GAM	
	Estimates	*p*-value	Estimates	*p*-value	Estimates	*p*-value	Estimates	*p*-value
Intercept	0.451	0.057	0.25	0.055	0.3853	0.0003***	0.597	0.009**
Arrested	0.677	<.001***	0.709	<.001***	0.677	<.001***	0.606	<.001***
Race	0.461	<.001***	0.584	<.001***	0.63	<.001***	0.523	<.001***
Drug	1.221	<.001***	1.183	<.001***	1.235	<.001***	1.066	<.0001***
TV time	0.007	0.027*	0.007	0.005**	0.008	<.001***	0.008	0.008**
Mistreatment	0.309	0.011**	0.293	0.003**	0.33	<.001***	0.214	0.07
Religion	-0.299	0.007***	-0.293	<.001***	-0.31	<.001***	-0.281	0.001***
Alcohol	0.541	<.001***	0.544	<.001***	0.577	<.001***	0.420	<.001***
Kid	0.208	0.017***	0.208	0.002**	0.245	<.001***	0.231	0.006**
Public work	-0.27	0.002**	-0.28	<.001***	-0.302	<.001***	-0.268	0.002**
Starting age	-0.006	<.001***	-0.006	<.001***	-0.007	<.001***	-0.007	<.001***
Education	-0.473	<.001***	-0.509	<.001***	-0.497	<.001***	-0.484	<.001***
Income	-0.074	<.001***	-0.234	<.001***	-0.073	<.001***	-0.087	<.001***
Gender	-0.144	0.1149	-0.17	0.031	-0.18	<.001***	-0.166	0.063
Sports	-0.486	<.001***	-0.493	<.001***	-0.436	<.001***	-0.47	<.001***
Insurance	-0.15	0.177	-0.189	0.029*	-0.158	0.002**	-0.144	0.186
Routine check	-0.111	0.207	-0.103	0.113	-0.11	0.006**	-0.078	0.369
Attitude future	-0.152	0.095	-0.16	0.046*	-0.152	0.001***	-0.143	0.112
Social relation	0.065	0.482	0.102	0.062	0.096	0.024**	0.058	0.526
Job	0.102	0.274	0.082	0.098	0.078	0.070	0.12	0.195
Covariance Parameters								
*var[Intercept]*	0.099	0.023	0.009	<.001***	N/A		N/A	
*var[Arrested]*	0.116	0.025	0.118	<.001***	N/A		N/A	
*cov[int & Slope]*	0.028	0.027	0.03	<.001***	N/A		N/A	

*p<0.05, **p<0.05, ***p<0.05

In general, we concluded that individuals who had higher income level were less likely to have smoked. People who had kids were about 1.23 times more likely to have smoked regularly. If one had alcohol or drug misuse, they were about 3.26 times more likely to smoke. Individuals who had been arrested or had spent more time watching TV or playing video games were about 1.96 times more likely to smoke than those with no such habits. Non-Caucasian were about 1.58 more likely to smoke than Caucasian. People not practicing religion were about 1.34 times more likely to smoke than those who were religious. People with lower education level were about more than 1.32 times likely to smoke. Individuals who drank alcohol early in life were more than 1.52 times more likely to be a regular smoker. People who experienced mistreatment from their parents during childhood were more than 1.70 times more likely to smoke regularly. Job satisfaction had no significant impact on smoking use. Finally, people who felt isolated from social activities had no impact.

### Results of standard logistic regression

As a point of reference regarding the extra variation or overdispersion present in the data due to its hierarchical nature, we fitted a standard logistic regression with 19 covariates. The goodness- of- fit test for overdispersion (Hosmer-Lemeshow, X^2^ = 19.97^,^ *p*-value = 0.011) suggested that the model is not a good fit and overdispersion is clearly present. This may be in part due to ignoring the hierarchical structure of the design. Initially, we fitted an unconditional logistic regression model with random effects (community) and no covariates and obtained an ICC value of 0.146. Thus, the intraclass correlation is large enough to suggest using a correlated model to address the hierarchical structure [12].

### Results of frequentist hierarchical and bayes’ hierarchical logistic regression model

A frequentist hierarchical logistic regression model with random intercept and random slope based on the differential rate due to the covariate *arrested*, resulted in the estimate of the variance for the intercept as 0.099 and random slope of 0.116 with its standard errors of 0.062 and 0.071 respectively. The covariance estimate between intercept and slope is 0.028. Thus, resulting in a standardized value of 0.099/0.062 for p-value of 0.023 for intercept and 0.116/0.071 for p-value of 0.025 and suggesting that the rate of change as it pertains to arrest varied across communities. Covariates, alcohol, kid, arrested, race, drug, TV time, mistreatment and religion showed significant positive effects on the probability of smoking, while covariates religion, public work, starting age, education, income, gender, sports showed significant negative effects on the probability of smoking. Thus, one expected that the probability that an individual who smoked was impacted by factors, although unidentifiable, but were related to the communities and history with the law as it depended on community. Mistreatment, gender, attitude towards the future, social relation and whether or not they had a job had no impact on the smoking behavior.

We fitted a Bayes’ hierarchical logistic regression model with random effects to measure in community random slope in arrested. A Bayes’ hierarchical logistic regression model fitted with MLWin gave random effects in community and arrested as significant with ${\displaystyle {\sigma}_{intercept}^{\overset{\frown }{2}}}=0.009$ and ${\displaystyle {\sigma}_{arrested}^{\overset{\frown }{2}}}=0.118$ and $\sigma \overset{\frown }{int/ arr} = 0.030$ with standard errors 0.002, 0.035, and 0.009, respectively. This suggested that our random effects due to community and due to arrest across communities differed significantly.

### Comparison of hierarchical models

The hierarchical models which tell about the conditional means (probabilities) gave similar results though for *social relation*, *routine check*, *insurance*, and *attitude towards the future* they differed. In particular, the models agreed with the set of covariates we categorized under demographics or health. When difference appeared they occurred mainly with Bayes’ and double GLM joint modeling model. In those cases, the Bayes’ hierarchical showed some marginal significance while the frequentist did not identify any trace of significance with these covariates. Bayes’ results have a tendency to have smaller *p*-values. This may be due to the fact that the Bayes’ method uses a prior distribution on the parameters.

### Results of joint modeling of mean and dispersion

A joint modeling of the mean and the dispersion with a generalized linear model for each submodel with covariate *arrest* in the dispersion sub-model provided a good fit to the data. The mean sub-model consisted of covariates, alcohol, kid, arrested, race, drug, TV time, mistreatment, social relation and job showing significant positive effects on the probability of smoking. The covariates religion, public work, starting age, education, income, insurance, sports, routine check, attitude future showed significant negative effects on the probability of smoking, Table 2.

The joint modeling of the mean and the dispersion with generalized additive submodels was fitted to the data. The smoothing effects were significant (*p* = 0.0001) with smallest GCV value of 3.96, based on estimating the additive predictors by using a B-spline smoother with 3 degrees of freedom including a strong quadratic pattern.

### Comparative dispersion submodel

In both the mean and the dispersion submodel for both the GLM and GAM, covariate arrest had a significant impact (*p* < 0.001) on the dispersion (see Table 3).

**Table 3:** Estimates of coefficients in dispersion submodel

	Joint GLMs		Joint GAMs	
Parameter	Estimate	*p*-value	Estimate	*p*-value
Intercept	-2.016	<.001	-0.519	<.001
Arrested	0.551	<.001	1.051	<.001

In the next section, we examined the predictive probability for each of these models based on the validation set and the training set in four different datasets. We used this in our comparisons as we took a closer look at the predictive ability of the models.

### Accuracy comparison of four models

We used the area under the Receiver Operating Characteristic (ROC) curves for both the training dataset and the validation dataset as a measure of fit for our four logistic regression models. We compared the four models based on their predicted probabilities in the training dataset and the validation dataset (see Table 4). We repeated the process four times, with a different 25% of the data omitted for validation each time. The joint modeling of the mean and the dispersion with GAMs had the best predicted probabilities among validation dataset. However, the four models did not show marked differences in their predicted probabilities.

**Table 4:** Accuracy Comparison of four models based on 4-fold

	ROC							
Method	Testing dataset	Validation dataset	Testing dataset	Validation dataset	Testing dataset	Validation dataset	Testing dataset	Validation dataset
Frequentist Hierarchical	0.7957	0.7375	0.7757	0.7608	0.7875	0.74	0.7795	0.7658
Bayes’ Hierarchical	0.7527	0.7279	0.7501	0.7377	0.7447	0.7228	0.7587	0.7500
Joint GLMs	0.7613	0.7378	0.7528	0.7613	0.7596	0.7411	0.75	0.7691
Joint GAMs	0.7769	0.7516	0.7671	0.7765	0.773	0.7594	0.7679	0.7769

## Discussion

We fitted four binary logistic regression models, frequentist hierarchical, Bayes’ hierarchical model, GLM joint modeling of mean and dispersion, and GAM joint modeling mean and dispersion to model smoking. Two of these models (the joint mean and dispersion submodel) addressed the marginal probabilities while the other two hierarchical models (Frequentist and Bayes’) addressed the conditional probabilities. The conditional probability models found *arrest* as a covariate with differential rates across communities while the marginal model also found that *arrest* is a key variable in explaining the dispersion. Overall, the models did not perform markedly different and gave similar results based on the training dataset and the validation dataset. The marginal models were similar with their predictability in the validation datasets and the training datasets.

We acknowledge the limitation of using a single dataset and encourage future research to conduct similar studies on a variety of correlated data. In the meantime, we are confident about the applicability of our conclusion to other research domains. Even though we did not perform a simulation study, we found similar patterns with hierarchical models with the UCLA dataset and recent research [12]. Similar results were also seen in the fit of the conditional models. The consistency of the models and the fit with validation and training datasets led us to believe in the generalizability of this paper to broader receivers.

## Conclusion

We fitted both conditional and marginal models to study smoking behavior for adolescents who had eventually become adults. While these models are addressing two different questions, we did not detect significant differences in both model performances based on training or validation. Further investigation showed that most communities were alike with a few showing that different random effect. By wave 4 these adolescents were moved away from their original communities so over time the community impact was negligent or independent of the smoking behavior.

## Additional file

Additional file 1: Appendix: SAS Code Used to Perform Models. (DOCX 150 kb)

## Abbreviations

Add Health: The National Longitudinal Study of Adolescent to Adult Health (Add Health)
COPD: Chronic obstructive pulmonary disease
DGLM: Double generalized linear models
GAM: Generalized additive model
GCV: Generalized cross validation
GEE: Generalized estimating equation
GLMM: Generalized linear mixed model
ICC: Intraclass correlation coefficient
MADAM: Mean and dispersion additive model
ROC: Receiver operating characteristic

## Footnotes

1 We define the ICC as the ratio of the variance component due to clusters to the total variance for individuals, such that $\mathrm{I}\mathrm{C}\mathrm{C} = \frac{{\displaystyle {\updelta}_{\mathrm{cluster}}^2}}{{\displaystyle {\updelta}_{i\mathrm{ndividual}}^2}+{\displaystyle {\updelta}_{\mathrm{cluster}}^2}}.$
