# Supplementary material for: Impact of communities, health, and emotional-related factors on smoking use: comparison of joint modeling of mean and dispersion and Bayes’ hierarchical models on add health survey
Source: BMC Med Res Methodol. 2017 Feb 3;17:20. doi: 10.1186/s12874-017-0303-y (PMC5291991; doi:10.1186/s12874-017-0303-y)
Supplement: Additional file 1: — Appendix: SAS Code Used to Perform Models. (DOCX 150 kb) [file 12874_2017_303_MOESM1_ESM.docx]

**Additionala file 1**

**SAS PROGRAM FOR STANDARD LOGISTIC REGRESSION AND ROC CURVES**

**proc** **logistic** data=final.train1;

class alcohol(ref='0') cluster2 smoking

married(ref='0') education(ref='0') income(ref='1') kid(ref='0') arrested(ref='0') gender(ref='0') white(ref='0')

insurance(ref='0') routine_check(ref='0') sports(ref='0') drug(ref='0') mistreatment(ref='0') job(ref='0')

mental(ref='0') religion(ref='0') public_work(ref='0') attitude_future(ref='0') parent_relation(ref='0') social_relation(ref='0')

income2(ref='0') income3(ref='0')/param=ref;

model smoking(EVENT = '1')= start_age alcohol

education incomes kid arrested gender white

insurance sports drug TVtime routine_check mistreatment

religion public_work attitude_future social_relation job/ LACKFIT outroc=troc;

output out=final.Toutput1 p=Tpred1;

score data=final.test1 out=final.Vpred1 outroc=vroc;

**run**;

**proc** **logistic** data=final.Toutput1 plots(only)=roc;

model smoking(event='1') = Tpred1;

**run**;

**proc** **logistic** data=final.Vpred1 plots(only)=roc;

model smoking(event='1') = P_1;

**run**;

**SAS PROGRAM FOR FREQUENTIST HIERARCHICAL LOGISTIC REGRESSION with VALIDATION**

**PROC** **GLIMMIX** DATA=final.train1;

CLASS alcohol(ref='0') cluster2 smoking

married(ref='0') education(ref='0') income(ref='1') kid(ref='0') arrested(ref='0') gender(ref='0') white(ref='0')

insurance(ref='0') routine_check(ref='0') sports(ref='0') drug(ref='0') mistreatment(ref='0') job(ref='0')

mental(ref='0') religion(ref='0') public_work(ref='0') attitude_future(ref='0') parent_relation(ref='0') social_relation(ref='0');

MODEL smoking(EVENT = '1')= start_age alcohol

education incomes kid arrested gender white

insurance sports drug TVtime routine_check mistreatment

religion public_work attitude_future social_relation job/DIST=BINARY LINK=LOGIT DDFM=BW SOLUTION;

RANDOM INTERCEPT arrested/ SUBJECT =cluster2;

OUTPUT OUT=final.Toutput3 pred(ilink)=Tpred3 resid=residual3 LCL=Tlower3 UCL=Tupper3;

store final.Score3; /* store the model */

**run**;

/* Get predictive data set */

**proc** **plm** source=final.Score3;

score data=final.test1 out=final.VPred3/ilink; **run**;

/* ROC curve for testing data set */

**proc** **logistic** data=final.Toutput3 plots(only)=roc;

model smoking(event='1') = Tpred3;

**run**;

/* ROC curve for validation data set */

**proc** **logistic** data=final.VPred3 plots(only)=roc;

model smoking(event='1') = predicted;

**run**;

*******************************************************************************************;

**proc** **glimmix** data=final.train2;

CLASS alcohol(ref='0') cluster2 smoking

married(ref='0') education(ref='0') income(ref='1') kid(ref='0') arrested(ref='0') gender(ref='0') white(ref='0')

insurance(ref='0') routine_check(ref='0') sports(ref='0') drug(ref='0') mistreatment(ref='0') job(ref='0')

mental(ref='0') religion(ref='0') public_work(ref='0') attitude_future(ref='0') parent_relation(ref='0') social_relation(ref='0');

MODEL smoking(EVENT = '1')= start_age alcohol

education incomes kid arrested gender white

insurance sports drug TVtime routine_check mistreatment

religion public_work attitude_future social_relation job/DIST=BINARY LINK=LOGIT DDFM=BW SOLUTION;

random intercept arrested/subject=cluster2;

OUTPUT OUT=final.Toutput4 pred(ilink)=Tpred4 resid=residual4 LCL=Tlower4 UCL=Tupper4;

store final.Score4;

**run**;

/* Get predictive data set */

**proc** **plm** source=final.Score4;

score data=final.test2 out=final.VPred4/ilink;

**run**;

/* ROC curve for testing data set */

**proc** **logistic** data=final.Toutput4 plots(only)=roc;

model smoking(event='1') = Tpred4;

**run**;

/* ROC curve for validation data set */

**proc** **logistic** data=final.VPred4 plots(only)=roc;

model smoking(event='1') = predicted;

**run**;

/* Calculating ICC */

SAS MACRO HPGLIMMIX FOR LOGISTIC REGRESSION MODELS WITH RANDOM SLOPE

title “ICC.slope.incomes”;

%***hpglimmix***(data=final.test2,

procopt=order=internal,

stmts=%str(

class smoking CLUSTER2 alcohol

married education income kid arrested gender white

insurance sports drug mistreatment

religion public_work attitude_future social_relation job;

model smoking = start_age alcohol

education incomes kid arrested gender white

insurance sports drug TVtime routine_check mistreatment

religion public_work attitude_future social_relation job/ s;

random intercept arrested/subject=cluster2;

),

error=binary,

link=logit,

tech=quanew

);

**run**;

/* %HPGLIMMIX macro */

/********************************************************************

%HPGLIMMIX: A SAS macro to fit generalized linear mixed model with

high dimensional fixed and/or random effects design matrix using

PROC HPMIXED and the Output Delivery System (ODS). This macro

is based on the %GLIMMIX macro (SAS v8.2) from SAS Institute, Inc

with PROC MIXED computing core replaced by PROC HPMIXED, as

well as many other modifications to comply with requirement

of PROC HPMIXED and other performance considerations. Currently

supported distributions include: BINARY, BINOMIAL, POISSON,

GAMMA, INVERSE GAUSSIAN, GEOMETRIC, and NORMAL (for reference only).

Requires SAS/STAT Version 9.2 or above.

Author : Liang Xie

History of %HPGLIMMIX

-------

initial stable version 21May2011 LX

Per suggestions from Laurence V. Madden

of OSU, corrected bugs when model

statement used PARMS statement 28Jan2012 LX

Corrected minor bugs found by Laurence V.

Madden from OSU 16May2012 LX

Corrected bugs on TEST statement found

by Laurence V. Madden of OSU 13Mar2013 LX

SAS CODE FOR DOUBLE GLM

%***doubleglm***(base=final.train2,

y=smoking,

x= start_age alcohol

education incomes kid arrested gender white

insurance sports drug TVtime routine_check mistreatment

religion public_work attitude_future social_relation job,

z= arrested,

dist=binomial,

link=logit,

intercept=y,

scale=deviance,

dist_disp=gamma,

link_disp=log,

intercept_disp=y,

alpha=**0.05**,

scale_disp=deviance,

maxiter=**100**, eps=**0.000001**, maxit=**50**);

***macro code for "doubleglm"; must follow

**data** final.Toutput6;

set final.train2;

Tlogithat = **0.381** -**0.006** * start_age + **0.5322** * alcohol -**0.4938** *education -**0.0771** * incomes + **0.2382** * kid

+**0.7061** * arrested -**0.0907** * gender + **0.6043** * white -**0.1646** * insurance -**0.4785** * sports

+**1.2165** * drug + **0.0074** * TVtime -**0.0864** * routine_check + **0.298** * mistreatment -**0.3155** * religion

-**0.2826** * public_work -**0.1805** * attitude_future + **0.0633** * social_relation + **0.1162** * job;

Tpred6=exp(Tlogithat)/(**1**+exp(Tlogithat));

**run**;

**data** final.Vpred6;

set final.test2;

Vlogithat = **0.381** -**0.006** * start_age + **0.5322** * alcohol -**0.4938** *education -**0.0771** * incomes + **0.2382** * kid

+**0.7061** * arrested -**0.0907** * gender + **0.6043** * white -**0.1646** * insurance -**0.4785** * sports

+**1.2165** * drug + **0.0074** * TVtime -**0.0864** * routine_check + **0.298** * mistreatment -**0.3155** * religion

-**0.2826** * public_work -**0.1805** * attitude_future + **0.0633** * social_relation + **0.1162** * job;

predicted=exp(Vlogithat)/(**1**+exp(Vlogithat));

**run**;

/* ROC curve for training data set */

**proc** **logistic** data=final.Toutput6 plots(only)=roc;

model smoking(event='1') = Tpred6;

**run**;

/* ROC curve for validation data set */

**proc** **logistic** data=final.VPred6 plots(only)=roc;

model smoking(event='1') = predicted;

**run**;

***********************************************************************************************************;

**SAS CODE FOR GAM**

**PROC** **GAM** data=final.train1 plots=components(commonaxes clm);

class alcohol married education income kid arrested gender white smoking

insurance sports drug mistreatment routine_check

religion public_work attitude_future social_relation job/descending;

model smoking(EVENT='1')=spline(start_age, df=**3**)

PARAM(alcohol incomes education kid arrested gender white

insurance sports drug TVtime routine_check mistreatment

religion public_work attitude_future social_relation job)/dist=BIN;

output out=final.Toutput7 RESIDUAL=res p=P_;

SCORE data=final.test1 out=final.Vpred7;

**run**;

**data** final.final_GAM_log_residual;

set final.Toutput7;

res2=resalcohol****2**;

log_res2=log(res2);

**run**;

**proc** **GAM** data = final.final_GAM_log_residual plots=components(commonaxes clm);

class alcohol married education income kid arrested gender white smoking

insurance sports drug mistreatment

religion public_work attitude_future social_relation job/descending;

model smoking(EVENT='1')=PARAM(arrested)/dist=BIN;

**run**;

/* ROC curve for testing data set */

**proc** **logistic** data=final.Toutput7 plots(only)=roc;

model smoking(event='1') = p_smoking;

**run**;

/* ROC curve for validation data set */

**proc** **logistic** data=final.Vpred7 plots(only)=roc;

model smoking(event='1') = p_smoking;

**run**;
